# Individual Differences in Delay Discounting Under Acute Stress: The Role of Trait Perceived Stress

**DOI:** 10.3389/fpsyg.2012.00251

**Published:** 2012-07-19

**Authors:** Karolina M. Lempert, Anthony J. Porcelli, Mauricio R. Delgado, Elizabeth Tricomi

**Affiliations:** ^1^Department of Psychology, New York UniversityNew York, NY, USA; ^2^Department of Psychology, Marquette UniversityMilwaukee, WI, USA; ^3^Department of Psychology, Rutgers UniversityNewark, NJ, USA

**Keywords:** delay discounting, stress, decision-making, future orientation, perceived stress

## Abstract

Delay discounting refers to the reduction of the value of a future reward as the delay to that reward increases. The rate at which individuals discount future rewards varies as a function of both individual and contextual differences, and high delay discounting rates have been linked with problematic behaviors, including drug abuse and gambling. The current study investigated the effects of acute anticipatory stress on delay discounting, while considering two important factors: individual perceptions of stress and whether the stressful situation is future-focused or present-focused. Half of the participants experienced acute stress by anticipating giving a videotaped speech. This stress was either future-oriented (speech about future job) or present-oriented (speech about physical appearance). They then performed a delay discounting task, in which they chose between smaller, immediate rewards, and larger, delayed rewards. Their scores on the Perceived Stress Scale were also collected. The way in which one appraises stressful situations interacts with acute stress to influence choices; under stressful conditions, delay discounting rate was highest in individuals with low trait perceived stress and lowest for individuals with high trait perceived stress. This result might be related to individual variation in reward responsiveness under stress. Furthermore, the time orientation of the task interacted with its stressfulness to affect the individual’s propensity to choose immediate rewards. These findings add to our understanding of the intermediary factors between stress and decision-making.

## Introduction

Delay discounting refers to the tendency for individuals to prefer immediate rewards over rewards received after a delay, even if the magnitude of the delayed reward is larger (Kirby et al., 1999; Berns et al., 2007). This tendency can often be maladaptive when making intertemporal choices; for example, one might choose to forego future health in order to enjoy the immediate pleasure afforded by fatty foods. While most individuals exhibit some degree of delay discounting, the rate at which people discount future rewards can vary widely from individual to individual, and even from context to context (Peters and Buchel, 2011). For instance, many studies have shown that drug addicts have higher discounting rates than non-drug users (Kirby et al., 1999; Kirby and Petry, 2004; Businelle et al., 2010), and people from Western cultures have higher discounting rates than those from Eastern cultures (Takahashi et al., 2009). Contextual influences on delay discounting rate include a gambling context among pathological gamblers (Dixon et al., 2006) and episodic future thinking in normal subjects (Peters and Buchel, 2010; Benoit et al., 2011).

Given that many important decisions are made under stressful circumstances, our aim in the present study is to determine the effect of acute stress on delay discounting rate. A few studies have begun to investigate how stress might influence decision-making about delayed rewards and uncertain rewards. Participants under stress have been shown to make more risky choices in a risk-taking paradigm (Porcelli and Delgado, 2009), and exogenous cortisol administration has been shown to increase risk-seeking (Putman et al., 2010). While these findings point to more risky decision-making under stress, which may translate to higher delay discounting, high basal cortisol levels have actually been associated with less risky behavior on the Iowa Gambling Task (van Honk et al., 2003), as well as with lower delay discounting rates (Takahashi, 2004). Stress has also been found to increase risky decision-making in a driving task in older adults, but not in younger adults (Mather et al., 2009), and a few studies have reported gender differences in decision-making under stress (Preston et al., 2007; Lighthall et al., 2009; van den Bos et al., 2009; Takahashi et al., 2010).

These mixed findings highlight the importance of considering not only the presence of an acute stressor, but also how people perceive and appraise stressful situations. A growing literature shows that individuals may interpret stressful situations as either challenging or threatening, and that this might affect their choices (e.g., Kassam et al., 2009). The degree to which an individual tends to perceive stressful situations as uncontrollable, unpredictable, and severe can be measured by the Perceived Stress Scale (PSS; Cohen et al., 1983). High scores on this measure have been associated with blunted hedonic capacity (Pizzagalli et al., 2007), especially in the presence of acute stress (Bogdan and Pizzagalli, 2006). This decreased reward responsiveness may affect the way in which immediate rewards are construed, and decisions are made, in a delay discounting paradigm. In the present study, we administered the PSS in order to measure participants’ general perception of life events as stressful. This trait perceived stress might represent an important intermediary factor in the relationship between acute stress and delay discounting; likewise, acute stress may mediate the relationship between trait perceived stress and delay discounting.

In addition to varying in the way they are interpreted by the individual, stressful situations may also vary in their time orientation. That is, they may be future-focused (e.g., thinking about future encounters or job interviews) or present-focused (e.g., worrying about how one appears to others). Since intertemporal choice involves making decisions about the future, and since perception of time is an important factor in determining delay discounting rates, it is critical to take into account how stress interacts with time orientation to affect decisions involving delayed rewards. Individuals who perceive future events as being farther away in time are more likely to discount rewards at a higher rate (Takahashi, 2005; Zauberman et al., 2009). In addition, prospection about future events has been found to reduce bias toward immediate reward in delay discounting tasks (Peters and Buchel, 2010; Benoit et al., 2011). Prospection about future events in a stressful context may have a different effect on delay discounting, in that a bleak view of the future might invite a preference for immediate reward. Likewise, stress that is present-focused may decrease delay discounting rate by decreasing responsiveness to immediate reward (Bogdan and Pizzagalli, 2006). In order to control for effects of time orientation on delay discounting, we included manipulations that varied in both stressfulness and time orientation.

Another important consideration is the distinction between risk-taking and delay discounting. While delay discounting carries a risk-taking component (since future rewards may be interpreted as uncertain), the inability to wait for future rewards is a well-documented and well-defined dimension of impulsivity (Kirby and Finch, 2010). Risk-taking is often found to be correlated with preference for immediate reward (Reynolds et al., 2004), and there may be similarities between the discounting functions for delayed and probabilistic rewards (Rachlin et al., 1991; Green and Myerson, 2004). However, probability discounting (i.e., tendency for risk-taking) and delay discounting likely have distinct neural underpinnings and mechanisms (Cardinal, 2006), and other variables may affect these two constructs differently (Green et al., 1999; Green and Myerson, 2004). Therefore, in order to examine whether stress affects delay discounting, probability discounting, or both, this study also included a choice paradigm in which participants made decisions between certain and probabilistic rewards. Higher probability discounting rates were indicative of greater risk-taking, or a greater propensity to discount odds against receiving an uncertain reward.

In summary, the current study explored the effects of acute stress on delay discounting rate and probability discounting rate, while taking into consideration individual differences in trait perceived stress and time orientation of the stressor. Each of four groups was treated with a different manipulation, designed to induce some combination of time orientation and acute stress. Additionally, PSS scores were collected for all participants. Due to previously discussed evidence of reduced reward responsiveness in high PSS individuals during acute stress exposure, we predicted that delay discounting rate under stressful conditions would be differentially affected in individuals with varying levels of trait perceived stress.

## Materials and Methods

### Participants

A total of 120 males participated in this experiment; they were randomly assigned to each of the four groups. Smokers were excluded, because smoking has been linked with high delay discounting rates (Businelle et al., 2010), as well as increased base cortisol levels (Kirschbaum et al., 1992). Only males were included because of reported menstrual and contraceptive effects on cortisol levels (Kudielka et al., 2004). Limiting analysis to one gender is a common approach in the stress and cognition literature (e.g., al’Absi et al., 2002; Henckens et al., 2009). In addition, the experiment was always conducted between the hours of 1:00 and 5:00 PM, as stress levels have been shown to fluctuate throughout the day (Izawa et al., 2010) and to control for circadian fluctuations in circulating cortisol (Federenko et al., 2004). Seven individuals were excluded from data analysis due to misunderstanding of instructions (*n* = 2), refusal to consent to video recording (*n* = 2), and naïveté issues (i.e., having done a similar stress-induction study previously; *n* = 3). Therefore, final analysis was conducted on 113 participants (mean age = 20.46 years, SD = 3.74). All participants gave written, informed consent. They were compensated with course credit, and all were aware that they might be selected to perform a stressful speech-giving exercise while being videotaped. The study was approved by the Institutional Review Board of Rutgers University.

### Working memory task

**Figure 1:**
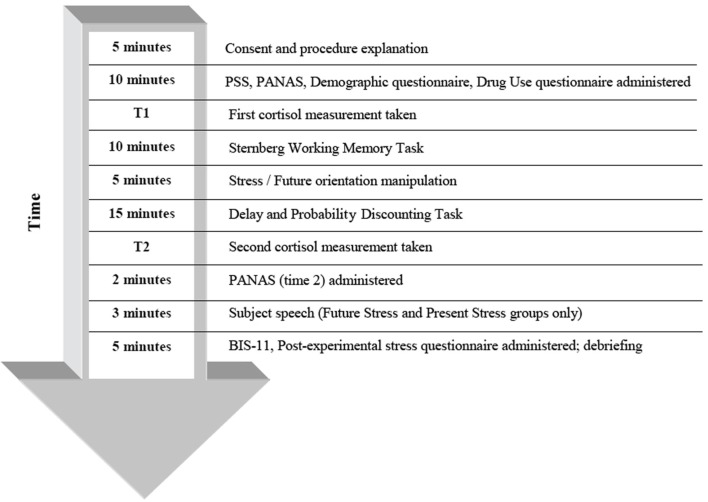
**Timeline for procedure**.

Prior research has demonstrated that lower working memory capacity may be related to increased delay discounting (Hinson et al., 2003; Shamosh et al., 2008; Bobova et al., 2009). Thus, we included a variant of the Sternberg item-recognition task (Sternberg, 1966), often used in past research to measure maintenance of information in verbal working memory (Rypma et al., 2002; Narayanan et al., 2005; Porcelli et al., 2008). Participants completed this task directly after a baseline cortisol measurement was taken (for study timeline, see Figure 1). On each of forty trials, subjects were presented with a string that was either three or six letters in length. After a brief pause, they were shown a single random letter and were asked to indicate whether or not this letter had appeared in the previous string by pressing “1” (for “yes”) or “2” (for “no”).

### Stress and future orientation manipulation

After the participants completed the working memory task, they were told whether or not they had been randomly selected to give a speech. In our factorial design, participants were exposed either to acute stress in the form of anticipating a videotaped speech or a non-stressful control procedure (Stress, Non-Stress). Additionally, participants’ time orientation was manipulated to be either future- or present-focused (Future, Present). Therefore, there were four distinct manipulations, detailed below, which varied on these two axes. The anticipatory stress manipulation was adapted from a previous study on stress and decision-making (Preston et al., 2007).

If the participants were in the future-oriented stress (FS) group, they were told that they would be giving a 3-min speech in front of a video camera. The speech would be recorded, and later viewed and judged by the experimenters. The instructions for the speech were, “Pretend that you are in front of an interview committee for your future dream job. Talk about your strengths and weaknesses, and please explain why you should get this job despite your weaknesses. You will be ranked relative to your peers based on your articulation, clarity, defensiveness, openness, and organization.” The participants were then given 5 min to prepare their speeches before the next task. This speech topic is commonly used as part of the Trier Social Stress Test (Kirschbaum et al., 1993), a manipulation that reliably elicits self-reported stress and cortisol release.

In the present-oriented stress (PS) group, the instructions were similar, but the topic of the speech was “Please talk about what you dislike about your body and physical appearance.” It was emphasized that participants should focus only on physical traits. Preston et al. (2007) found that this speech topic elicited stress in both men and women.

Both stress conditions included a similar speech-giving exercise that involved some level of social evaluative stress. We chose these procedures because their validity and effectiveness in inducing stress has been demonstrated in previous studies (e.g., Kirschbaum et al., 1993; Preston et al., 2007). Although time orientation was not the only factor that differed between the two conditions, we did not want to make the time orientation manipulation transparent to subjects, nor did we wish to include a new manipulation that might not have been effective in inducing stress.

In the future-oriented non-stress (FN) group, participants were told to relax and to make a list of events within the next 6 months that they were looking forward to (e.g., school vacations).

If the participants were randomly selected to be in the present-oriented non-stress (PN) group, they were asked to relax and sit quietly while listening to music (Winston, 1984, track 4). Listening to relaxing music is often used as a non-stressful control task in studies that involve stress manipulation (e.g., van den Bos et al., 2009).

### Delay and probability discounting task

The delay and probability discounting task administered following the stress and time orientation manipulations was a computerized question-based measure used in past research to study choice behavior (Richards et al., 1999). In the series of delay trials, participants were presented with questions asking about their preferences between $10 to be received after one of the delays (1, 2, 30, 180, and 365 days) or a smaller amount (e.g., $2) to be received immediately. For each trial, they were instructed to click on the reward they preferred. Time to choose was unlimited, and after each response participants were asked, “Are you sure about your response?” If they indicated uncertainty, they were permitted to go back to make a different choice; if they were sure, the program continued to the next question. For each delay, the immediate reward amount was increased or decreased in value (±$0.50) based on previous responses until an indifference value was reached. An indifference value is defined as the smallest amount of money chosen to be received immediately instead of waiting the specified delay in order to receive the $10 standard. A random adjusting-amount procedure was programmed to use the answers to previous questions to restrict the range of values from which the immediate value for the next question was selected. This procedure was unlikely to be transparent to participants, because delay trials were interspersed with probability trials (see next section), and the algorithm for determining the adjusted value for subsequent questions was not readily predictable. No participant indicated that he detected the adjusting nature of the task.

Interspersed with delay trials were probability trials. In these trials, participants were presented with a choice between $10 to be received at varying levels of probability (25, 50, 75, and 90%) or a smaller amount with a 100% chance of receipt. As in the delay trials, the smaller amount was increased or decreased in value (±$0.50) based on previous responses until an indifference value was reached. The initial adjusted value was randomized, and the range of potential indifference values was restricted with every response. An indifference value for each probability was defined as the smallest amount of money chosen to be received with certainty instead of taking the probabilistic $10. The task automatically terminated once an indifference value was calculated for each delay and for each probability (for more details on this procedure, see Richards et al., 1999).

To increase the saliency and relevance of their choices, participants were told at the outset that one of their responses would be randomly selected and that they would receive the amount they chose at the delay specified, or with the probability specified. That is, if they chose the immediate reward on the randomly selected trial, they would receive the money as additional compensation after the session; conversely, if they chose the larger, delayed reward, they would receive the money either by mail, or by returning to the laboratory, after the delay specified. If the randomly selected trial was a probability trial, they would either receive a reward at the end of the session (if they chose the “definite” option) or they would draw a token from a bag containing two colors of tokens in the proportion that reflected the probability of their selection, thereby possibly receiving compensation at the end of the session. After completing this procedure, participants were asked to rate their certainties for receiving the delayed rewards (e.g., “If you had chosen the money delayed by 365 days, were you sure you would actually get that money if it was the randomly selected answer? How sure were you that you would get the money in 365 days if you chose it?”; Richards et al., 1999).

Following this task, salivary cortisol was sampled (T2). Participants in the stress condition were then asked to give the 3-min speech, while participants in the non-stress condition were informed of their winnings in the delay discounting task. Participants in the stress condition were not informed about their winnings until after the speech.

### Cortisol

Salivary free cortisol levels correspond well with plasma free cortisol levels. Therefore, collection of saliva is an easy and non-invasive means to obtain an index of the biologically active fraction of this hormone (Kirschbaum and Hellhammer, 1989). Participants were instructed to refrain from eating and consuming caffeine and alcohol for at least 2 h before study participation. Saliva samples were collected using Salimetrics Oral Swabs (SOS) after the first set of questionnaires (T1) and approximately 20 min after the stress manipulation (T2). Cortisol levels corresponding to the stress response tend to peak at this time (Kirschbaum et al., 1993). Subjects chewed on the SOS for about 60 s, after which they were placed in vials and stored in a freezer until later processing. Cortisol samples were assayed at Salimetrics, LLC (State College, PA, USA).

### Self-report questionnaires

Self-report questionnaires administered at the beginning of the experiment included the PSS (Cohen et al., 1983) and the Positive and Negative Affect Schedule (PANAS; Watson et al., 1988). The PSS measures the degree to which situations in one’s life are appraised as stressful (Cohen et al., 1983). Higher scores indicate higher perceived stress in response to stressful situations. The PANAS contains items that measure current negative affect and positive affect. High negative affect is characterized by subjective distress, nervousness, and overall unpleasant engagement. Positive affect, on the other hand, represents the degree to which the individual engages with the environment with positive emotions, such as enthusiasm and alertness (Watson et al., 1988). Demographic and income information were also collected at this time, and drug and alcohol use were assessed. The first cortisol measurement was taken as soon as these questionnaires were completed (T1).

Directly after the delay discounting task, participants completed the PANAS one more time. At the end of the experiment, the Barratt Impulsiveness Scale Version 11 (BIS-11; Patton et al., 1995) and a post-task questionnaire assessing stress during the experiment were administered. The BIS-11 provides a measure of real-world impulsivity (e.g., “I charge more than I earn”) and has been found to possess satisfactory reliability and validity (Patton et al., 1995).

## Results

### Data reduction

The indifference values determined in the delay discounting task were used to produce a delay discounting curve and to compute the area under the curve (AUC; Myerson et al., 2001) for each subject. The indifference values and delay values (1, 2, 30, 180, and 365 days) were used as *x*-coordinates and *y*-coordinates, respectively, to construct a graph of the discounting data. Vertical lines were then drawn from each data point to the *x*-axis, subdividing the graph into a series of trapezoids. The area of each trapezoid is equal to (*x*_2_ − *x*_1_)[(*y*_1_ + *y*_2_)/2], where *x*_1_ and *x*_2_ are successive delays, and *y*_1_ and *y*_2_ are the indifference values associated with these delays (for the first trapezoid, the value of *x*_1_ and *y*_1_ are defined as 0 and 1). The area under the empirical discounting function is equal to the sum of the areas of these trapezoids.

Area under the curve values range from 0 to 1; higher AUC values indicate lower discounting by delay (i.e., a preference for delayed, larger rewards), while lower AUC values correspond to steeper, or more impatient, discounting (i.e., a preference for smaller, more immediate rewards). The AUC served as the measure of delay discounting rate in this study. As a theoretically neutral measure of delay discounting, the AUC makes no explicit assumptions regarding the form of the indifference curve. In this way, it is applicable to a wider range of indifference curves than other quantitative models, such as hyperbolic or exponential delay discounting models. This approach is common in studies of delay discounting (e.g., Dixon et al., 2006; Shiels et al., 2009).

For the probability choice data, AUC was also computed (Myerson et al., 2001), using indifference values for each probability as the *y*-coordinate, and the odds against receiving a reward as the *x*-coordinate. AUC was used as the measure of probability discounting rate. Here, smaller area values indicate greater discounting by odds against receiving a reward; therefore, participants with lower AUC values were relatively risk-averse, while higher AUC values indicated greater risk-taking behavior.

We tested for Pearson correlation between participants’ scores on the working memory measure and delay discounting rate and probability discounting rate, as well as between BIS-11 scores and these variables. No significant relationships were found between working memory capacity and delay discounting rate (*r* = 0.06, *p* = 0.55) or probability discounting rate (*r* = 0.08, *p* = 0.41). There were also no significant associations between scores on the BIS-11 and delay discounting (*r* = −0.05, *p* = 0.64) or probability discounting (*r* = 0.01, *p* = 0.96). Therefore, these variables were dropped from further analyses.

### Effects of acute stress manipulation

Two participants’ cortisol data were excluded due to sample contamination, leaving 111 subjects in this analysis. There was an effect of acute stress manipulation on change in cortisol from T1 to T2, with stress groups showing significantly greater increases in cortisol than non-stress groups [*F*_(1, 110)_ = 5.17, *p* = 0.025; change in cortisol for future stress group: *M* = 0.005 μg/dL, SD = 0.116 μg/dL, range: −0.28 to 0.28 μg/dL; for present stress group: *M* = −0.003 μg/dL, SD = 0.073 μg/dL, range: −0.19 to 0.14 μg/dL; for present non-stress group: *M* = −0.04 μg/dL, SD = 0.068 μg/dL, range: −0.2 to 0.17 μg/dL; for future non-stress group: *M* = −0.028 μg/dL, SD = 0.053 μg/dL, range: −0.19 to 0.06 μg/dL]. There was also a main effect of stress manipulation group on change in negative affect from T1 to T2 [*F*_(1, 110)_ = 8.99, *p* = 0.003], indicating presence of greater negative affect after the stress manipulation than before it. There was no significant relationship between change in negative affect and change in cortisol, neither across all subjects (*r* = 0.05, *p* = 0.60) nor for participants in the stress groups (*r* = −0.04, *p* = 0.80). Not all participants completed the post-task questionnaire, assessing the stressfulness of the manipulation; therefore, this explicit measure was not considered in the manipulation check.

Between the two stress groups, FS and PS, no differences in cortisol (*t*_54_ = 0.29, *p* = 0.77,) or negative affect (*t*_55_ = 1.09, *p* = 0.28) were observed, implying that participants in these groups exhibited similar stress reactivity associated with the two manipulations. The groups did not differ with regard to changes in positive affect (*t*_55_ = 1.23, *p* = 0.23) from pre- to post-manipulation. Furthermore, there were no differences in change in positive (*t*_54_ = −0.15, *p* = 0.89) or negative affect (*t*_54_ = −0.93, *p* = 0.36) from T1 to T2 between the two non-stress groups, PN and FN, demonstrating that they were similarly emotionally influenced by the non-stress manipulations.

### Acute stress interacts with trait perceived stress to affect delay discounting

**Figure 2:**
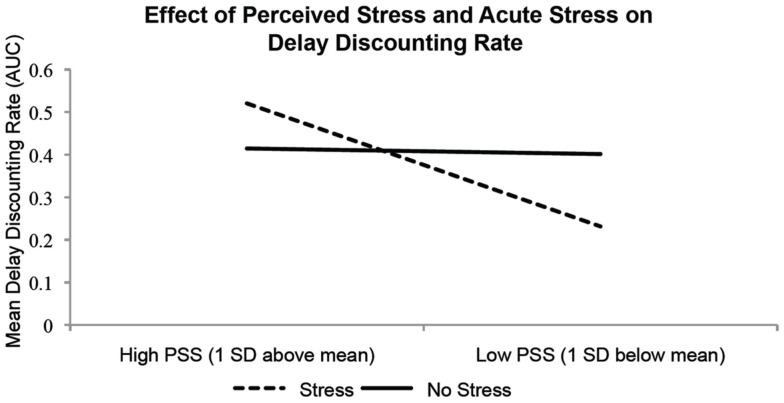
**Mean delay discounting rates for individuals with PSS scores 1 standard deviation above and below the mean, stratified by stress manipulation group (Stress, Non-stress; *N* = 113)**. There is a significant interaction of acute stress manipulation and trait perceived stress level on delay discounting rate (AUC). Higher AUC values indicate lower delay discounting rates (i.e., more later/larger rewards chosen). When no stress is present, participants with differences in trait perceived stress make similar choices on the delay discounting paradigm. When faced with an acute stressor, subjects with high PSS scores discount rewards at a lower rate, and subjects with lower PSS scores exhibit increased discounting.

To investigate how individual differences in trait perceived stress might interact with acute stress to determine decision-making patterns, a moderated multiple regression approach was used (Aguinis, 2004). A one-way ANOVA indicated that PSS did not significantly vary by group (*F* = 0.315, *p* = 0.814). Mean-centered PSS scores, the dummy-coded acute stress exposure variable (non-stress = comparison group), and the dummy-coded time orientation variable (present = comparison group) were entered into the first step of the regression, with the interaction term between PSS score and acute stress manipulation entered in the second step. Delay discounting rate and probability discounting rate were entered as dependent variables in two separate analyses. This analysis showed that the interaction between acute stress and PSS significantly predicted delay discounting rate [PSS range: 8–43, *M* = 23.56, SD = 6.57; Δ*R*^2^ = 0.06, Δ*F*_(1, 108)_ = 6.71, *p* = 0.016; Figure 2], better than the model that included PSS, acute stress exposure, and time orientation of the stressor. When including certainty about receiving delayed rewards (as assessed by the post-task questionnaire) as another control variable in the first step of this analysis, the interaction remains significant (Δ*R*^2^ = 0.06, Δ*F*_(1, 107)_ = 5.29, *p* = 0.007). Delay discounting was highest (i.e., AUC value is lowest) in individuals with low trait perceived stress when they were under acute stress, and lowest for individuals with high trait perceived stress when they were under acute stress. In the first step of this regression, a model containing mean-centered PSS scores and the dummy-coded acute stress exposure and time orientation variables was not sufficient to predict delay discounting rate (*R*^2^ = 0.05, *F*_(1, 109)_ = 1.80, *p* = 0.15). The multiple regression analysis revealed null results when probability discounting rate was entered as a dependent variable [Δ*R*^2^ = 0.01, Δ*F*_(1, 108)_ = 0.230, *p* = 0.19].

As a confirmatory analysis, a three-way ANOVA was performed, with Time Orientation (Future, Present), Acute Stress (Stress, Non-Stress), and median-split PSS score (High PSS, Low PSS) as factors, and with delay discounting rate as the dependent variable. There were no significant main effects of any of the factors, but the interaction between PSS score group and acute stress group was significant [*F*_(1, 112)_ = 5.188, *p* = 0.025].

### Effect of time orientation and acute stress on immediate reward bias

In a 2 × 2 ANOVA, the effects of stress [*F*_(1, 112)_ = 1.36, *p* = 0.25] and time orientation manipulation on choices about probabilistic rewards in this paradigm did not reach significance [although there was a trend for future orientation; *F*_(1, 112)_ = 3.63, *p* = 0.06]. There was no overall significant effect of future orientation on delay discounting rate [*F*_(1, 112)_ = 0.37, *p* = 0.55], nor was there an overall effect of acute stress [*F*_(1, 112)_ = 0.55, *p* = 0.46]. All interactions were also non-significant [for probability discounting: *F*_(1, 112)_ = 1.25, *p* = 0.27; for delay discounting: *F*_(1, 112)_ = 0.41, *p* = 0.53].

Due to evidence that future-thinking manipulations can decrease delay discounting rate (e.g., Benoit et al., 2011), the lack of a significant relationship between time orientation and delay discounting rate in this study was puzzling. Thus, in an additional analysis, we quantified participants’ *immediate reward bias*, by calculating the choice index, or the ratio of the frequency of immediate reward options chosen to all options chosen (Boettiger et al., 2007; Benoit et al., 2011) for each subject. This immediate reward bias measure is distinct from the delay discounting rate, since it reflects participants’ overarching preference for immediate reward, regardless of the delay intervals. Past studies (e.g., Ebert and Prelec, 2007) have shown that manipulations of time sensitivity may affect choices for the near-future and far-future differently, thereby changing delay discounting in a way that might not be captured by the AUC measure.

**Figure 3:**
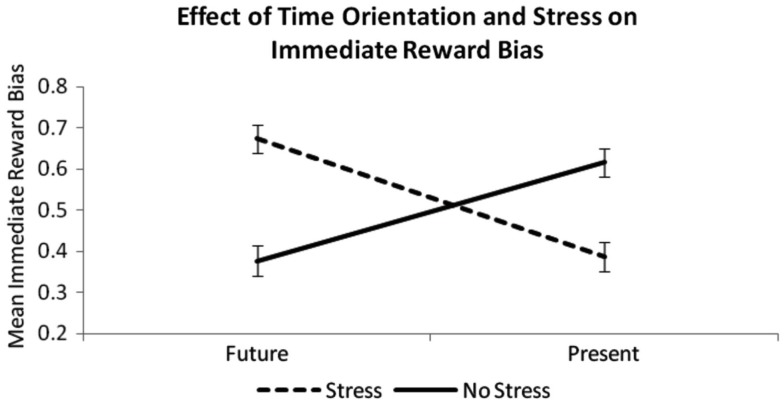
**Means of immediate reward bias (i.e., percentage of immediate options chosen) for each group (Future Stress, Future Non-stress, Present Stress, Present Non-stress)**. There is a significant interaction between time orientation manipulation and acute stress manipulation on this variable [*N* = 112; *F*_(1, 111)_ = 56.63, *p* < 0.001], showing that future orientation increases choices for immediate reward when the stressor is present, but decreases immediate reward bias when the stressor is absent.

A 2 × 2 ANOVA was conducted with Time Orientation (Future, Present) and Acute Stress (Stress, Non-Stress) as factors and immediate reward bias as the dependent variable. No significant main effect of future orientation on the percentage of immediate reward choices was observed [*F*_(1, 112)_ = 0.47, *p* = 0.50], nor was there a main effect of acute stress [*F*_(1, 112)_ = 0.88, *p* = 0.35]. However, there was a significant interaction between time orientation manipulation and stress manipulation [*F*_(1, 112)_ = 56.63, *p* < 0.001; Figure 3], whereby immediate reward bias was highest when a future-oriented situation was stressful (FS) or a present-oriented situation was non-stressful (PN), and it was lowest when a future-oriented situation was non-stressful (FN) or a present-oriented situation was stressful (PS). *Post hoc* *t*-tests revealed that the PN group exhibited significantly higher immediate reward bias than the PS group (*t*_55_ = 4.44, *p* < 0.001) and the FN group (*t*_54_ = 4.61, *p* < 0.001). In addition, the FS group demonstrated higher immediate reward bias than the PS group (*t*_55_ = 6.10, *p* < 0.001) and the FN group (*t*_54_ = 6.34, *p* < 0.001). There were no differences between the FS and PN groups in immediate reward bias (*t*_52_ = −0.16, *p* = 0.87), nor was there a significant difference between the FN and PS groups on this measure (*t*_55_ = 1.18, *p* = 0.24). The ANOVA remains significant even after entering certainty about receiving future rewards as a covariate [*F*_(1, 111)_ = 55.82, *p* < 0.001]. However, we found that participants in the PS group were significantly less certain that they would receive a reward after 365 days than members of the other groups (compared to FN: *t* = −2.63, *p* < 0.05; FS: *t* = −2.09, *p* < 0.05; PN: *t* = −2.04, *p* < 0.05). This difference between groups is worthy of note.

This finding should be interpreted with caution, as the percentage of immediate rewards chosen was strongly correlated with the number of choices that participants faced in this staircase paradigm (*r* = 0.609, *p* < 0.001). Indeed, if the first immediate reward amount randomly generated by the task was very high, then it would have been more likely to be followed by more immediate reward choices; if the first number was low, then there would be fewer immediate choices at the beginning. This source of noise is potentially problematic, but due to its random nature, it is unlikely to be at the root of group differences. It is plausible, however, that another mechanism (such as more consistent choices among subjects in the FN and PS groups) may be driving this effect. To investigate this possibility further, we fit a *q*-exponential model to the data to determine estimates of *q* (inconsistency) and *k_q_* (delay discounting rate) using R statistical language (www.r-project.org). This function is based on Tsallis’ statistics, and was computed as:

(1) $$V\left(D\right)={A∕\left[\text{1 + }\left(\text{1}-q\right)k_{q}D\right]}^{1∕\left(1-q\right)}$$

where *A* is the amount of the reward at *D*, *D* is the delay to reward, *k_q_* is a parameter of delay discounting at delay *D*, and the *q* parameter can be used to assess a subject’s consistency in intertemporal choice (Cajueiro, 2006; Takahashi et al., 2007). Note that Eq. 1 is equivalent to the simple hyperbolic discount function [*V*(*D*) = *A*/(1 + *kD*)] when *q* = 0. Critically, in the current dataset, the q-exponential function could not be fitted in 26 participants due to an infinity value. After excluding these subjects, a one-way ANOVA revealed no group differences in the consistency parameter *q* (*F* = 0.513; *p* = 0.679) or in the discounting parameter *k_q_* (*F* = 0.487; *p* = 0.692). The lack of quantifiable differences in consistency between groups indicates that this finding most likely arises from a change in bias toward immediate reward. However, in this staircase procedure, it is important to bear in mind that the immediate reward bias index is a less reliable measure than AUC.

### Effect of cortisol on delay discounting

Collapsing across all groups, there was a significant negative correlation between change in cortisol concentration from T1 to T2 and delay discounting AUC (*r* = −0.19; *p* < 0.05), whereby higher discounting of delayed rewards (smaller AUC) was associated with a larger increase in cortisol after the manipulation. That is, individuals who experienced an increase in cortisol, regardless of manipulation, were more likely to select smaller, sooner rewards. When inspecting the stress groups only, however, this relationship does not hold (*r* = −0.16, *p* = 0. 23), although the direction of the effect is the same. There was no significant relationship between change in cortisol and probability discounting (*r* = −0.06; *p* = 0.512), or with immediate reward bias (*r* = 0.14; *p* = 0.16).

## Discussion

Many decisions are made under stressful circumstances, including decisions in which small, immediate rewards are weighed against larger, delayed rewards. The results of the present study carry strong implications regarding the effects of acute stress on intertemporal choice. In this investigation, we found that the interaction between trait perceived stress and acute stress had a significant effect on rate of delay discounting, regardless of the time orientation of the stressor. Based on our findings, we can conclude that individuals with high and low trait perceived stress made different choices when faced with acute stress. Those who are more likely to perceive stressful situations as such show a preference for larger, delayed rewards, while those with low perceived stress discount delayed rewards at a higher rate. When there was no acute stressor present, individuals who differed in PSS levels made similar choices in this paradigm. It is possible, then, that individual differences in stress appraisal may affect reward responsiveness under stress. When these same analyses were performed on probabilistic choice data, null effects were observed.

The finding that choices under stress were differentially affected by *a priori* level of trait perceived stress speaks to the complexity of stress as a construct, and the importance of studying individual differences in this domain. The challenge versus threat literature on stress (Blascovich and Tomaka, 1996; see also Henry, 1980; Frankenhaeuser, 1986) differentiates “good stress” from “bad stress” during active, goal-relevant tasks. Whether an individual perceives a stressful situation as a challenge or a threat may affect the decisions that one makes in such a situation (e.g., Kassam et al., 2009). In addition, the perceived controllability of a stressor can influence executive functioning under stress (Henderson et al., 2012). A plausible mechanism in the current study is that those who are more likely to perceive situations as stressful are more likely to interpret the stress manipulation as threatening. Accordingly, they might experience a decrease in their reward response to immediate reward and make more delayed reward choices. This decrease in reward responsiveness has been documented in previous studies (Bogdan and Pizzagalli, 2006), and the association between blunted reward response and reduced delay discounting has also been found previously (Lempert and Pizzagalli, 2010). Those with low trait perceived stress, on the other hand, may feel more control over the stressor, and even see the situation as a challenge with a positive valence. Thus, they may experience an increased immediate reward response and choose more immediate rewards. In addition, high trait perceived stress has been shown to be hereditary (Bogdan and Pizzagalli, 2009) and associated with a serotonin transporter genotype that is linked with depression following stress (Otte et al., 2007). Given serotonin’s role in delay discounting processes (Schweighofer et al., 2008), the interplay between serotonin levels, stress, and decision-making is an interesting future avenue of research.

In this study, we also found that the interaction between time orientation and acute stress had a significant effect on bias toward immediate reward. Participants tended to make far-sighted choices when they experienced present stress or when they thought about the future in a stress-free light. Conversely, when participants thought about a future situation that was stressful (in this case, a future job interview), they showed a greater preference for immediate – albeit smaller – rewards. These findings suggest that induction of a future orientation is not sufficient to reduce delay discounting rate. Past studies that have found an effect of prospection on discounting rate (Peters and Buchel, 2010; Benoit et al., 2011) focused only on positive future events. Framing a future situation as stressful, however, might precipitate a bleak view of the future, which, in turn, shifts a participant’s motivation toward increasing immediate reward. Changes in mood might be involved in this process (Hirsh et al., 2010; Augustine and Larsen, 2011). In our study, it is impossible to disentangle effects of mood from effects of stress; in fact, negative affect increased significantly more for all participants who underwent a stress manipulation relative to those in the non-stress conditions.

Another methodological limitation of our study is that there might be differences between our future non-stress and present non-stress groups independent of time orientation itself (e.g., arousal state may be different for listing positive future events than for listening to music). Similarly, there may be differences between the stress groups that are unrelated to time orientation; in the present stress condition, participants are socially evaluated for more superficial qualities (physical appearance), whereas in the future stress condition, they are evaluated for deeper qualities. Although there were no significant differences between the two stress conditions in cortisol increase, participants may have also felt more stressed in the present stress condition, due to the uncontrollability of the speech topic (subjects had more freedom to choose a topic in the “future job” speech condition). The aim of the current study was to investigate the effects of acute stress on delay discounting, while controlling for the potential confound of time orientation. Therefore, statistical differences in decision-making based on time orientation should be interpreted with caution. Future studies are warranted to further clarify the contributions of time orientation and stress to delay discounting.

While future orientation and stress, in combination, affected immediate reward bias, they did not have any effect on *rate* of delay discounting. This finding is unusual, given that these two variables were derived from the same choice procedure, and that they are correlated (*r* = −0.604, *p* < 0.01). However, the percentage of small-immediate rewards chosen does not fully represent an individual’s tendency to choose more proximal rewards versus more distal ones. Only delay discounting rate takes into account the various delays used in the paradigm, which ranged from 1 to 365 days. It is possible that certain manipulations, such as our future orientation manipulation, may induce an ephemeral tendency toward choosing either immediate or future rewards, without affecting the more stable variable of discounting rate, characterized in this study by AUC. For example, Ebert and Prelec (2007) found that manipulations of time sensitivity affected the valuation of near-future and far-future rewards differently.

With our AUC measure of delay discounting, it is not clear if the differences reported above are due to effects on the discount parameter (i.e., to what degree are sooner rewards valued more than later rewards), or effects on the participants’ utility functions (i.e., how the objective reward amounts correspond to participants’ subjective values). Previous studies have found that delay discounting behavior can be explained by a combination of diminishing marginal utility and preference for sooner reward (Andersen et al., 2008; Pine et al., 2009). It is also possible that our manipulations influenced time perception in these subjects, which then modulated their delay discounting (Takahashi, 2005; Zauberman et al., 2009). Future research will be necessary to clarify the effects of stress on time perception, marginal utility, and time discounting.

Stress hormones, such as cortisol, are known to influence a number of brain regions related to decision-making. They seem to impair prefrontal cortex (PFC) function and executive control (Hains and Arnsten, 2008), but they activate different receptors in PFC depending on the level of stress, and depending on the time of day (see Lupien et al., 2007 for a review). One limitation of the current study is that, even though we always conducted the study in the afternoon, we did not assess participants’ sleep habits. Acute stress might affect glucocorticoid activity in early risers and late-risers differently; furthermore, late-risers and early risers may differ in their decision-making patterns (e.g., Tonetti et al., 2010). However, all participants in the study were students, and most had similar class schedules, so it is unlikely that their sleeping patterns varied widely. Stress hormones can also impair hippocampal function and neurogenesis (McEwen, 1999). White matter volume in the hippocampus has been shown to be positively associated with delay discounting rate (Yu, 2012), and the hippocampus is involved in future-directed thinking during delay discounting (Peters and Buchel, 2010). Determining the relationship between stress, hippocampal activity, and delay discounting is a promising avenue for future research.

Unlike previous studies on stress and decision-making (e.g., Porcelli and Delgado, 2009), we found no significant effect of acute stress on choices in the probability portion of the decision-making paradigm. However, differences in experimental procedures, including stress application and actual paradigm, might be responsible for this discrepancy. In contrast with many risk-taking tasks, in our task, participants made decisions about a large range of probabilities under no time pressure. There were also no correlations found between delay discounting and working memory capacity or BIS-11 scores, but these null findings are unsurprising, since this study examined manipulations of delay discounting, and not trait delay discounting. That is, any correlations across all participants may have been overwhelmed by larger, between-group differences.

The inclusion of males only in this study can be seen as both a strength and a limitation. While it is a standard practice in stress research, and we were able to rule out gender effects on the hormonal data, gender differences have been observed in studies that utilize stress manipulations (Preston et al., 2007; Lighthall et al., 2009; van den Bos et al., 2009; Takahashi et al., 2010). Because gender is an important variable of interest in studies of stress and decision-making, we felt that excluding its consideration in this already complex study was warranted. It is premature, however, to generalize our results about the effects of stress on decision-making in this sample to the general population; future studies should aim to uncover gender differences if they exist.

The present study provides important evidence that a general outlook toward stress can affect decision-making under stress. This finding is relevant to the prevention of substance dependence and other disorders of impulsivity (e.g., attention deficit/hyperactivity disorder, pathological gambling). Both high delay discounting (Yoon et al., 2007; Harty et al., 2011) and stress (Sinha, 2001) have been shown to be vulnerability factors for addiction and relapse. It has also been hypothesized that stress might elicit suicidal behavior and other impulsive behaviors in depression, through modulation of delay discounting (Takahashi, 2011). By addressing how individuals handle stress, and manipulating the way that immediate rewards are perceived under stress, it may be possible to intervene in the development of maladaptive and dangerous behaviors.

In conclusion, the presence of acute stress interacts with general perceived stress to influence discounting of delayed rewards. Furthermore, future orientation and acute anticipatory stress show interactive effects on bias toward immediate reward. Whether one is contemplating the present or the future during intertemporal choice affects the likelihood with which one chooses immediate rewards, but this effect is tempered by the stressfulness of the context. Countless crucial decisions are made under stress every day. The current findings add to our understanding of the mediating factors that act between acute stress and decision-making.

## Conflict of Interest Statement

The authors declare that the research was conducted in the absence of any commercial or financial relationships that could be construed as a potential conflict of interest.
